# Risk Factors for Zoonotic Tuberculosis at the Wildlife–Livestock–Human Interface in South Africa

**DOI:** 10.3390/pathogens8030101

**Published:** 2019-07-14

**Authors:** Petronillah R. Sichewo, Anita L. Michel, Jolly Musoke, Eric M.C. Etter

**Affiliations:** 1 Department of Veterinary Tropical Diseases, Bovine Tuberculosis and Brucellosis Research Programme, Faculty of Veterinary Sciences, University of Pretoria, Private Bag X04, Onderstepoort 0110, Pretoria, South Africa; 2 Department of Animal and Wildlife Sciences, Faculty of Natural Resources Management and Agriculture, Midlands State University, P. Bag 9055, Gweru, Midlands 00263, Zimbabwe; 3 Research Associate at the National Zoological Gardens of South Africa, Pretoria 0001, South Africa; 4 National Health Laboratory Services, Department of Medical Microbiology, Universitas, Bloemfontein 9301, South Africa; 5 Department of Medical Microbiology, Faculty of Health Science, University of the Free State, Bloemfontein 9301, South Africa; 6 Department of Production Animal Studies, Faculty of Veterinary Sciences, University of Pretoria, Private Bag X04, Onderstepoort 0110, South Africa; 7 CIRAD, UMR Animal, Santé, Territoires, Risque et Ecosystèmes (ASTRE), 34398 Montpellier, France; 8 ASTRE, Univ Montpellier, CIRAD, INRA, 34398 Montpellier, France

**Keywords:** bovine tuberculosis (bTB), multiple correspondence analysis (MCA), *Mycobacterium bovis* (*M. bovis*), risk factors, wildlife–livestock–human interface, zoonotic TB

## Abstract

A cross-sectional study was conducted to investigate the risk factors associated with zoonotic tuberculosis in humans and its transmission to people living at the wildlife–livestock–human interface. A questionnaire was administered to collect information on food consumption habits, food handling practices, and knowledge of zoonotic TB. Sputum samples were also collected from 150 individuals that belonged to households of cattle farmers with or without a bTB infected herd. In addition, 30 milk samples and 99 nasal swabs were randomly collected from cattle in bTB infected herds for isolation of *Mycobacterium bovis* (*M. bovis*). The sputum samples were screened for TB using the GeneXpert test and this was followed by mycobacterial culture and speciation using molecular techniques. No *M. bovis* was isolated from TB positive sputum samples and only one sample was confirmed as *Mycobacterium tuberculosis* (*M. tuberculosis*). *M. bovis* was isolated from 6.6% (n = 2/30) milk samples and 9% (n = 9/99) of nasal swabs. Ownership of a bTB infected herd and consumption of milk were recognized as highly significant risk factors associated with a history of TB in the household using multiple correspondence analysis (MCA) and logistic regression. The findings from this study have confirmed the potential for zoonotic TB transmission via both unpasteurized milk and aerosol thus, the role of *M. bovis* in human TB remains a concern for vulnerable communities.

## 1. Introduction

Cattle are the maintenance host of Mycobacterium bovis (*M. bovis*) but many other domestic and wildlife animals can be affected by the pathogen [1]. *M. bovis* belongs to the Mycobacterium tuberculosis complex (MTC), a group of closely related organisms that causes TB in mammals including humans [2]. Although *M. tuberculosis* is the most common cause of human TB cases, an unspecified fraction occurs due to *M. bovis* infection and is referred to as zoonotic TB [3,4,5].

The true impact of *M. bovis* on the human TB epidemic is unclear due to the lack of routine bovine TB surveillance data from humans and resources for the identification of *M. bovis* [6,7]. In 2016 it was estimated that 147,000 new cases of zoonotic TB were reported in people, and 12,500 deaths due to the disease globally and most of these occurred in Africa [8]. Using the data that was available at the time of the study, Müller et al. (2013) estimated that *M. bovis* might be responsible for up to 37.7% of all human TB cases in Africa [6]. In studies carried out in several African countries *M. bovis* has been isolated from lymph node biopsies or aspirates of TB patients, in Uganda at a prevalence of 7%, in Tanzania 16% and in Ethiopia 17% [9,10,11]. While recent investigations in Zambia and Uganda diagnosed *M. bovis* at a prevalence of < 1% and < 3%, respectively from sputum samples of pulmonary TB patients [12,13].

Zoonotic TB is primarily acquired through the consumption of unpasteurized milk and dairy products, less frequently from eating of raw or improperly cooked infected meat and via aerosols inhaled from infected animals during direct human–livestock contact [5,14]. This supports the observation that zoonotic TB occurs more frequently as extra-pulmonary TB (EPTB) (9.4%) in humans than in the pulmonary form (2.1%) as mentioned by Etter et. al., (2006) [15]. The ‘test and slaughter policy’, compulsory pasteurization of milk and abattoir surveillance have been successfully implemented in developed countries leading to the near elimination of the disease in cattle and human populations [6]. Unlike in developing countries, particularly in Africa, these policies are absent or inadequate due to lack of resources, thus bTB is widespread in animals [16].

Risk factors for *M. bovis* transmission to people include demographic factors (e.g., number of family members, age), feeding habits, people living in close contact with their animals, socio-economic status, illiteracy (lack of knowledge of zoonotic TB), customs, traditions coupled with the increasing prevalence of HIV/AIDS pandemic [5,14,16,17]. Nevertheless, the principal risk factors that govern bTB epidemiology and transmission to humans living at the wildlife–livestock–human interface remain largely unknown in sub-Saharan Africa including South Africa.

In northern KwaZulu-Natal, in addition of bTB being endemic in wildlife in the game reserves such as the Hluhluwe iMfolozi, the disease was previously detected in cattle in the surrounding communal area with an overall herd prevalence of 28% [18,19]. The community is in this province with one of the highest TB incidence rate of 685/100,000) and an HIV prevalence of 27% in the country, where consumption of unboiled milk is a common practice, indicative of a significant role of *M. bovis* in human TB [20,21,22]. In addition, there is a presumptive lack of knowledge among livestock farmers on zoonotic TB and protective practices against zoonotic TB transmission have never been examined in the area. Against this background, there was a need to investigate the possible risk factors for bovine tuberculosis transmission from cattle to humans in a One Health approach and to assess the farmers’ knowledge of zoonotic TB. The findings from this study will assist the policy makers in the selection of appropriate preventive animal health measures and designing of awareness programs led by community health workers.

## 2. Results

### 2.1. Household Study

Sputum and serum samples were collected from 75 individuals from households that owned bTB test positive cattle, similarly 75 samples were collected from households that owned bTB test negative cattle. This was less than the expected sample size of 300 and we attribute the low numbers to the absence of some family members during the times we visited their homesteads, and the unwillingness of either individuals or guardians (for children below the age of 15) to participate. Most of the individuals that participated were mostly men 62% (94/150) aged between 16–64 years as shown in Table 1. According to the cultural practices in the area, it is mostly the men that are involved in livestock activities.

*M. bovis* was not isolated from individuals in the study population. Out of the 10 samples that were TB positive from the GeneXpert test none of the samples were identified as *M. bovis*; only one sample was confirmed as *M. tuberculosis* through culture and deletion analysis of the RD4 and the rest of the samples were negative for mycobacterial organisms as shown in Table 1. The estimated population prevalence of *M. bovis* was 1.33% confidence interval (C.I) [0;3.92] and < 3.92% respectively for participants from households that owned bTB positive and uninfected herds. The GeneXpert test is the standard TB diagnostic in South Africa as recommended by the World Health Organisation for diagnosis of pulmonary TB [23]. The Gene Xpert (Cepheid), identifies *Mycobacterium tuberculosis* complex organism and determines drug resistance to rifampicin in TB positive samples. The overall HIV prevalence was 36% (individuals aged between 16–64) and there was no significant difference in HIV infection between the farmers that owned bTB infected and uninfected herds (Chi-squared test: *p* value = 0.5). Results in Table 1 summarizes the TB and HIV results from the household study. The participants that were unaware of their TB status (6%) or HIV status (10%) prior to enrolling in this study were informed of their results by the Department of Health at their respective clinics and received appropriate treatment.

### 2.2. Bovine Samples

A total of 30 milk samples and 99 nasal swabs were collected from bTB infected herds. The milk yield was low, and some lactating animals had ceased milk production due to the drought in the study area at the time of sampling. *M. bovis* was isolated from 9% (n = 9) of nasal swabs from infected herds and from 6.6% (n = 2) of the collected milk samples.

### 2.3. Questionnaire Survey

A total of 71 participants took part in the questionnaire survey, comprising 59% (n = 42) from households with bTB infected herds and 41% (n = 29) from households that owned uninfected herds. This is less than the calculated number of farmers (100) and this was due to the unavailability of family members because of work commitments and livestock herding activities. In a few cases there were elderly or young people at home who could not answer questions logically and people not willing to participate in the study.

### 2.4. Descriptive Analysis

Of the 71 participants, 40% (n = 31) reported a history of TB diagnosis in the family and 60% (n = 40) had no history of TB diagnosis in the family. The household demographics are summarized in Table 2 below.

Most of the participants were involved in the herding (100%) and milking of cows (86%). The participants reported consumption of milk (98%), especially as sour milk (89%) and this involved all the family members in 97% of the households. Most of the households obtained meat from their own cattle (94%) but none involved veterinary services for the inspection of the meat. More than 50% of the households had observed abnormal spots on organs that looked like TB lesions (as shown on the pictures by the interviewer) at least once during informal slaughtering. The family members of cattle owning households displayed poor knowledge of the disease transmission modes with 56% being unaware of the zoonotic aspect of bovine tuberculosis and 63% being unable to give examples of protective practices during slaughter of animals. This is summarized in Table 3.

### 2.5. Risk Factor Analysis

The analysis of risk factors was done using the history of TB in the family as reference variable (MCA) or the fixed (outcome) variable (Fisher test and general linear model).

#### 2.5.1. Univariate Analysis and Logistic Regression of Risk Factors for bTB Transmission to Humans

Out of the 20 variables that were analysed using univariate analysis, five significant factors were identified (*p* value < 0.25) namely: consumption of milk weekly and when needed, consumption of meat from own cattle or from other farmers and ownership of a bTB positive herd as shown in Table 4.

#### 2.5.2. Multiple Correspondence Analysis

**Figure 1:**
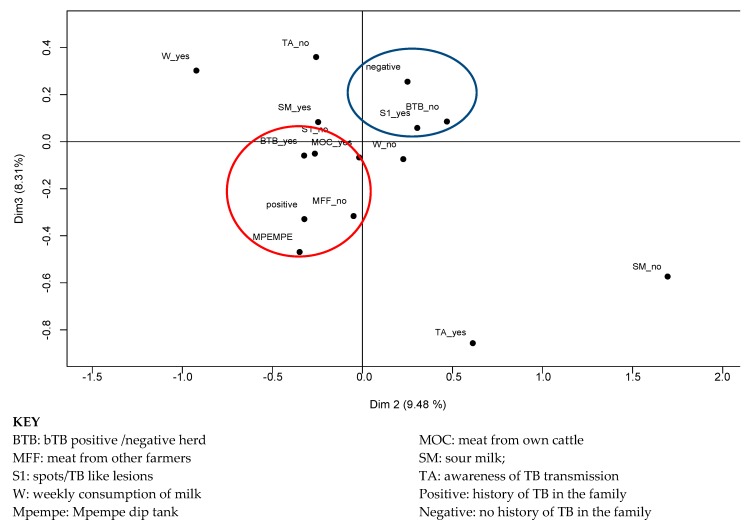
Multiple correspondence analysis of risk factors associated with history of TB diagnosis in the family (positive or negative).

The risk factors/practices that were closely related to the history of TB diagnosis in a family are: ownership of a bTB positive herd, not purchasing meat from other farmers, ownership of herds that belonged to Mpempe dip tank and farmers that had never seen abnormalities or TB like lesions on cattle organs during slaughter. Absence of history of TB diagnosis in the family was closely related to the ownership of an uninfected herd and previous observation of abnormalities or TB like lesions on organs from their animals. This is illustrated in Figure 1 depicting the history of TB diagnosis (positive) and related risk factors/practices inside the red circle and absence of history of TB diagnosis (negative) in the family and related protective factors/practices inside the blue circle.

#### 2.5.3. Logistic Regression of Risk Factors for bTB Transmission to Humans

All the statistically significant factors from the univariate analysis (*p* < 0.3) and other risk factors of biologically importance were included in the logistic regression model; the significant risk factors identified were, ownership of a bTB positive herd and consumption of milk from own cattle while consumption of meat from own cattle or other farmers were identified as protective practices as presented in Table 5.

## 3. Discussion

The aim of this study was to investigate *M. bovis* infection in humans exposed to bTB infected cattle and the risk factors associated with zoonotic TB in a community living at the wildlife–livestock–human interface. *M. bovis* excretion was confirmed in milk (6.6%) and nasal discharges (9%) and the associated risk factors identified included consumption of unpasteurized milk particularly as sour milk and ownership of bTB infected cattle. The actual excretion rate in milk and nasal discharges should, however, be deemed higher due to loss of viable organisms during sample processing, the intermittent shedding of the pathogen by infected animals and the sensitivity of the culture method [24,25].

Despite the community’s apparent exposure risk, *M. bovis* was not detected in sputum samples collected from individuals that belonged to households that owned cattle. This raises the question whether *M. bovis* is not transmitted in high enough numbers to infect humans or whether the loss of live organisms was too severe and reduced the viable bacterial concentration below the limit of detection, or alternatively whether the threshold of infection in cattle was too low to cause disease in humans. The latter is because bTB prevalence in the cattle populations under study was reported as 12% while the bTB prevalence in cattle in Britain, which was 40% in the 1940s, was linked to a 6% *M. bovis* infection burden in human TB [18,26,27]. Similar results have also been reported in Ethiopia at a wildlife–livestock–human interface with low bTB prevalence rate in cattle [28]. Another possible explanation for the absence of *M. bovis* infection in humans might be due to life-long protection against *M. bovis* as induced by Bacillus Calmette-Guerin (BCG) vaccination at birth. However, further evaluations have revealed that BCG’s protective efficacy against *M. tuberculosis* infection and progression to disease is not absolute [29]. It has also been proposed that humans are more resistant to *M. bovis* as compared to *M. tuberculosis* as explained by reduced transmissibility and a lower risk of human disease establishment after infection [27,30].

Considering consumption of milk was identified as a significant risk factor and the apparent excretion of *M. bovis* in milk by lactating animals, non-pulmonary TB is highly likely to affect the exposed individuals. Therefore, sputum might have been an unsuitable sample for the detection of *M. bovis* in people that might be affected by non-pulmonary forms of TB as discussed by Debi et al. 2014 [31]. On the other hand, the participants had no apparent TB clinical signs such as a productive chronic cough, thus they experienced it challenging to produce sputum and instead inappropriate samples of saliva were collected which could have reduced the chance of detecting an underlying *M. bovis* infection. This was also supported by the discrepancy in the GeneXpert and culture results whereby, 10 sputum samples were GeneXpert positive and yet only one sample was positive for *M. tuberculosis* using culture. The GeneXpert test is a PCR based test that depends on the pathogen’s DNA in saliva hence displays a higher sensitivity and specificity than culture [32], whereas, culture results are affected by the number and viability of bacteria and quality of the sputum processed [33] which was influenced by the distance and transport delays between collection and processing of sputum as the study was in a remote area.

In corroboration with our results, a study carried out in the Serengeti ecosystem of Tanzania did not find *M. bovis* from the sputa of TB patients despite the presence of the infection in cattle and wildlife [34]. Similarly, studies in Brazil, Uganda and Cote d’Ivoire found no *M. bovis* from human samples that included sputa despite bTB being prevalent in the cattle populations of these countries [12,35,36]. In contrast, *M. bovis* has been detected in sputum samples of livestock traders in Nigeria (10%) and pastoral communities in Ethiopia (15%) from both sputum and fine needle aspirates specimens [37,38]. The high prevalence in Ethiopia could be explained by cattle farmers sharing shelter with their livestock while in this study, none of the farmers shared living space with animals [17,38]. Findings from these individual studies indicate that zoonotic TB is a disease of public health importance particularly in poor-resourced communities that should not be ignored [6].

In this study, consumption of milk and meat from own cattle were identified as highly significant risk factors for TB in people. Moreover, most of the households (89%) reported consumption of milk as sour milk by almost everyone in the households (97%), predisposing the individuals to *M. bovis* infection. Consumption of soured milk frequently (weekly), as indicated by two thirds of the respondents may increase the risk of repeated *M. bovis* exposure for a higher number of consumers. It has been demonstrated that *M. bovis* persists for up to 14 days in soured milk depending on the initial bacterial concentration in raw milk, souring and storage temperature. This is further exacerbated by the commonly practiced pooling and supplementation of milk with left over sour milk “stock” for a continuous production of sour milk that results in the contamination of milk from uninfected animals [39]. The results suggest ingestion of contaminated food as the most important route of infection and this would primarily result in extra-pulmonary TB, making it highly unlikely to detect *M. bovis* infection in sputa. *M. bovis* infection is frequently associated with extra-pulmonary as demonstrated by studies in Tanzania and Ethiopia where *M. bovis* was more prevalent in cases of extra-pulmonary TB than pulmonary TB [10,38]. However, in the present study cases of lymphadenitis (EPTB) were not encountered. The initial *M. bovis* load from the lactating cows might be too low to survive souring hence absence of EPTB in people as supported by the absence of the bacteria from the culture of milk samples that were collected sporadically from the infected cows in the study area over a period of 12 months [40].

All the farmers reported absence of meat inspection by veterinary public health officers during slaughter and 48% had no knowledge of the TB lesions that characterised organs of an infected animal. Inspection of meat at slaughter is of significance to control the spread of *M. bovis* infection from infected cattle to humans through removal of contaminated organs or condemnation of carcasses with disseminated bTB. It also facilitates the trace-back of *M. bovis* infection to herd level in eradication programs such as the one that was implemented in European countries in the 1960s to control zoonotic TB in humans [27]. Consumption of meat from the supermarket that undergoes regular inspection at the abattoirs was reported by more than half of the families (58%). In addition, consumption of meat from own cattle or bought from other farmers were indicated as protective practices. The consumption of undercooked contaminated meat has been previously reported in other investigations as a potential risk factor for transmission of Mycobacteria and other zoonotic diseases to humans although the public health significance is yet to be quantified [5,14,41,42,43].

The households that owned uninfected herds were associated with the absence of previous diagnosis of human TB but also with reporting of previous observations of organ abnormalities in slaughter animals. Equipped with this knowledge these households may be less likely to consume infected meat and thus be better protected against food borne zoonoses. These findings support the assumption that knowledge of bTB in cattle and its control is linked to a reduced risk to zoonotic TB.

Previous studies have reported ownership of cattle, direct contact with animals and living in close proximity with animals as important drivers of zoonotic TB transmission to people [43,44,45]. In this study, livestock keeping activities were not identified as risk factors although all the respondents participated in at least one livestock keeping activity such as milking, herding or examination of animals, none of them slept with or near their animals. We conclude that transmission of *M. bovis* through direct contact was less likely to occur in our study area due to the short periods of exposure of individuals in a confined environment to infected herds. In contrast, the livestock traders of Nigeria trade in a congested environment for long periods of time with increased human-to-cattle contact leading to zoonotic TB [37].

Approximately two thirds of the respondents were not aware of zoonotic TB transmission from cattle to humans. The lack of understanding of disease transmission precludes protective practices that contribute to effective disease control programs. In contrast with other studies elsewhere which demonstrated a positive correlation of zoonotic TB knowledge with post primary education this was apparently not the case in our study where 59% of the participants had post primary education [43,46].

## 4. Materials and Methods

### 4.1. Study Area

The study was conducted from August to September 2017, in four villages from Big 5 False Bay Municipality in uMkhanyakude district, northern Kwa-Zulu Natal province, South Africa. The municipality is situated between game reserves that include iSimangaliso Wetland Park (formerly St. Lucia) and Hluhluwe iMfolozi Park (HiP). Most of the land in the municipality is used for subsistence farming, game lodge activities and the north-eastern parts of the municipality are occupied by densely settled rural traditional communities. The study involved the cattle owners from four villages (Mnqobokasi, Makhasa, Mduku and Nibela), that were associated with the four dip tanks respectively (Masakeni, Mpempe, Nkomo, Nibela) where bTB testing of cattle had been carried out as part of a research project in 2016 and 2017 [18].

### 4.2. Study Population

The study population was recruited from the households that owned cattle herds that belonged to one of the 4 dip tanks. Participation in the study was voluntary, participants were approached through the community health care givers and the purpose of the study was explained. The case-control study involved two groups of households; 50 households that owned bTB infected herds and an equal number of cattle farmers that owned uninfected herds. Three family members were recruited from each household representing the three age categories: adolescents below 15 years, adults 16–64 years and the elderly above 64 years. The total number of participants calculated was 300, comprising of individuals that included head of households (cattle owners), herd boys/man/women (cattle keepers) and female family members of the cattle owning households.

### 4.3. Household Study

At household level samples (sputum and serum) were collected from three members of the family and a questionnaire was administered to one member of the household, either the cattle owner or cattle keeper or female household member.

#### 4.3.1. Collection and Processing of Human Samples

The research team visited each of the selected households and collected two sputum samples of approximately 2–5 mL into sterile, leak proof plastic container and 3–6 mL of blood into serum separating tubes (SST) from each participant. The name, age and gender for each participant was recorded but for the purpose of the study and confidentiality a unique code was allocated to each participant and served as an identifier on the sample containers. The samples were transported to the local district hospital (Mseleni NHLS laboratory) in a cold chain at 4 ℃ for the GeneXpert test.

**Figure 2:**
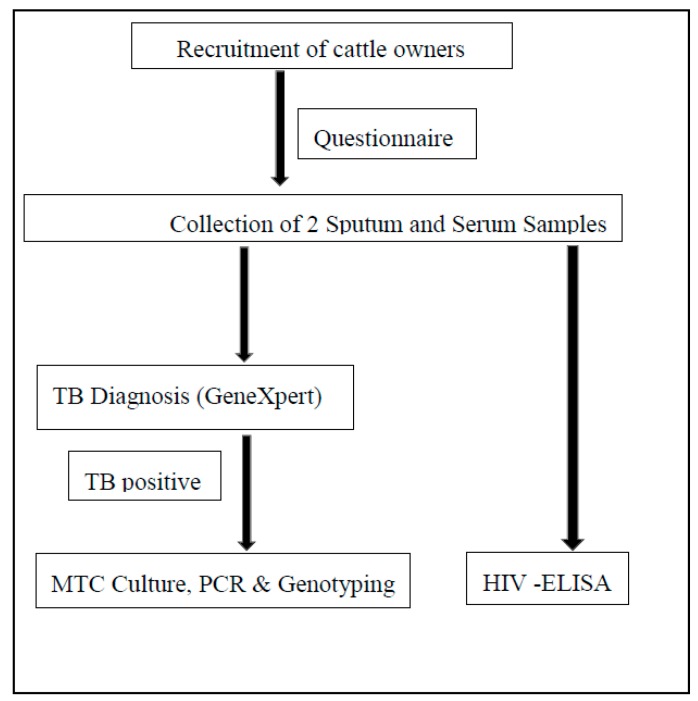
A flow chart of sample collection from households of cattle farmers (including cattle owners and cattle keepers) and laboratory processing for human samples (sputum and serum) and questionnaire administration.

The GeneXpert test is the standard TB diagnostic in South Africa as recommended by the World Health Organisation for diagnosis of pulmonary TB [23]. The Gene Xpert (Cepheid), identifies *Mycobacterium tuberculosis* complex organism (does not differentiate *M. bovis* and *M. tuberculosis*) and determines drug resistance to rifampicin in TB positive samples. The 2nd sputum samples from all the TB positive samples were transported in equal volumes of cetylpyridinium chloride (CPC) (1%) in a cold chain for mycobacterial culture and *M. bovis* identification at the National Health Laboratory Services (NHLS) Medical Microbiology University of the Free State. The serum samples were transported to Inkosi Albert Luthuli hospital-NHLS laboratory for HIV testing using the Enzyme-linked immunosorbent assay (ELISA) serological test. The sample collection and processing procedure is shown in Figure 2.

#### 4.3.2. Mycobacterium Species Culture and Identification

Sputum samples from participants found to be positive for TB during the screening test were inoculated into Mycobacterial growth indicator tube (MGIT) broth, incubated at 37 °C in a BD Bactec MGIT 320 with daily monitoring for 28 days and then once a week for up to 42 days. MGIT broth supports the growth of both *M. bovis* and *M. tuberculosis*. DNA was extracted from growth positive cultures using the Hain Genolyse kit reagents according to the manufacturer’s instructions. Briefly, 1 mL of the MGIT culture was centrifuged at 1000 g for 15 min, the pellet resuspended in 100 uL of lysis buffer followed by incubation for 5 min at 95 °C. For neutralisation, 100 uL of the buffer was added, centrifuged for 5 min at maximum speed and the supernatant stored as DNA for subsequent reactions. The DNA was used as a template in deletion analysis using PCR reaction for confirmation of mycobacterial growth as *M. bovis* targeting the region of difference (RD), RD4 as previously outlined by Warren et al. (2006) [47].

#### 4.3.3. Questionnaire Survey

A pre-tested questionnaire with open-ended and closed questions was administered for 20–30 min (face-to-face interviews) to one household member above 18 years that was involved with livestock keeping activities. The questionnaire was developed in English and was translated into the local language of isi-Zulu. The questionnaire was divided into four sections. The first section detailed demographic characteristics, and this included age, sex, level of education and number of family members. The second section prompted the interviewee to respond to questions pertaining to livestock management activities such as frequency of human-to-cattle contact during activities such as herding, milking and examination of the animals. The third section invited the respondents to answer questions on their food consumption behavior, food handling and processing practices. The questions in the fourth section were on the knowledge and awareness of bovine tuberculosis in cattle, humans and the history of TB in the family for the past 12 months.

### 4.4. Collection of Cattle Samples

Nasal swabs and milk samples were collected from cattle from bTB infected herds as previously determined by the interferon gamma assay (IFN-γ) (BOVIGAM^®^) [18]. Households with bTB positive herds brought their animals to the dip tank, milk was systematically collected from lactating animals and nasal swabs through random sampling of the animals. From a herd with >10 animals, every 5th animal was sampled while for herds < 10 every 3rd animal was sampled.

#### 4.4.1. Collection and Processing of Milk Samples

Verbal consent was obtained from the cattle owners before milking commenced. We intended to collect 50 mL of milk per animal, but the actual quantities collected depended on the availability of milk from the lactating animals. The milking was done manually, and 20–40 mL of milk was collected into sterile screw-capped 50 mL sterile centrifuge tubes by the cattle owner or cattle keeper after rinsing their hands and udder with 70% alcohol. The tubes were labelled with a unique code that identified the herd and placed in a cool box for transportation to the local state veterinary laboratories where it was stored at −20 °C until processing at University of Pretoria-Department of Veterinary Tropical Diseases BSL 2+ laboratory.

Milk decontamination was done using cetylpyridinium chloride (CPC) as previously described by Michel et al. [39]. The sediments were inoculated onto Löwenstein Jensen slopes supplemented with pyruvate and antibiotics cocktail (polymyxin B (200 IU/ml), amphotericin B (10 µg/ml) carbenicillin (100 µg/ml) and trimethoprim (10 µg/ml)) and incubated at 37 °C for 10 weeks with weekly monitoring for bacterial growth.

#### 4.4.2. Collection and Processing of Nasal Swabs

Nasal swabs were collected from bTB infected cattle using a 50-cm long homemade sterile swab made of aluminum wire and gauze from one nasal passage per animal. These were immediately immersed and expunged into 25 mL of sterile phosphate buffer saline (PBS) at pH = 7 in 50 mL centrifuge tubes in the field. The samples were kept at 4 °C until they were processed for *M. bovis* isolation and identification. The decontamination of nasal swabs was performed using 2% HCl as described by Gcebe et al. [48]. The sediments obtained after decontamination were incubated for 20 hours after adding 1 mL of 50 µg/ml of amphotericin B (antifungal). The solution was then inoculated onto Löwenstein Jensen media with pyruvate and a cocktail of antibiotics (polymyxin B (200 IU/ml), amphotericin B (10 µg/ml) carbenicillin (100 µg/ml) and trimethoprim (10 µg/ml) (NHLS, South Africa)) and incubated at 37 °C for 10 weeks.

#### 4.4.3. Mycobacterium Bovis Identification

Crude DNA extraction from *M. bovis* isolates was done by boiling a loopful of cells in 100 µl of distilled water using a heating block for 25 min at 95 °C [49]. *M. bovis* was confirmed in the isolates using deletion analysis as described by Warren et al., targeting RD4 and RD9 primers recognized by the amplification of DNA products with band sizes 268 bp and 108 bp, respectively [47].

### 4.5. Statistical Analysis

All the data collected during the household study, questionnaire survey, bovine and milk samples results were stored in Microsoft Excel. The data from the questionnaire survey were exported and analysed with the R software (© 3.4.4, 2018, R Foundation for Statistical Computing, Vienna, Austria). Bovine TB transmission from cattle to humans was initially examined using general descriptive analysis. The effect of potential risk factors on the variable history of TB diagnosis in members of cattle owning households was analysed using the univariate analysis by means of the two-tailed Fischer’s exact test. The association of explanatory variables with a history of TB patients in the households was further described using the multiple correspondence analysis (MCA) [50]. The effect of potential risk factors on TB in people was analysed using univariate analysis using the two-tailed Fischer’s exact test. Logistic regression analysis was performed to quantify these risk factors (Generalised Linear model (GLM-family = binomial) using the significant risk factors from the univariate analysis (*p* < 0.25).

### 4.6. Ethical Clearance

Animal ethical and biosafety transport clearances were obtained from the University of Pretoria Animal Ethics committee (Ref: V078-16) and the Department of Agriculture Forestry and Fisheries under Section 20 (12/11/1/1/6/1). Ethical clearance for human study was obtained from the University of Pretoria Ethics committees in the Faculty of Health Sciences (321/2016) for sputum and sera collection and Faculty of Humanities (GWO170814HS) for the questionnaire survey. The study was also explained to the participants before collection of samples and verbal and written consent obtained.

## 5. Conclusions

The isolation of *M. bovis* from milk and nasal secretions confirms that there is a potential risk of bovine tuberculosis transmission from cattle to humans through exposure to respiratory exudates, from aerosols and supported by the consumption of contaminated raw and sour milk. Nevertheless, the current study could not confirm *M. bovis* transmission between animals and humans at the wildlife–livestock–human interface. Therefore, in this population there was no link between food consumption practices and transmission of zoonotic TB to people. However, the presence of *M. bovis* in animal products that are known to be consumed by farmers without specific precaution is of significance in the designing of control and management measures that will reduce the impact of the disease on human health.

## Figures and Tables

**Table 1:** *Mycobacterium tuberculosis* complex and HIV status of household members based on bTB herd status.

bTB Herd Status	Age Group	No. of Participants	TB Positive (GeneXpert)	Confirmed *M.tuberculosis*	HIV Prevalence
bTB positive herd	16–64 years	68	5	1	40% (30/75)
	>64 years	7	2	-	-
bTB negative herd	16–64 years	72	3	-	33% (25/75)
	>64 years	3	-	-	-

**Table 2:** Summary of household demographics determined by questionnaire survey.

Variable	Level	Response (n = 71) %
Gender	Male	66
	Female	34
Age group	16–64 years	87
	>64 years	13
Status in the family	Cattle owner	31
	Cattle keeper	11
	Member of the household	58
Education	No Education	18
	Primary education	23
	Secondary education	17
	High school	34
	Tertiary	8

**Table 3:** Risk factors and awareness of bTB as determined by questionnaire.

Category	Variable	Level	Responses (n = 71) %
Food consumption habits	Do you regularly consume milk from your animals?	Yes	98
		No	2
	Who mainly consumes milk in the household?	Whole household	97
		<12	1.5
		>64 years	1.5
	How often is milk consumed?	Daily	3
		Weekly	63
		When needed	34
	How do you consume your milk?	Soured (Amasi)	89
		Raw	6
		Boiled	4
	What is your source of meat?	Supermarket	58
		Own cattle	94
		Buy from others	17
	How do you process meat with abnormal spots?	Never seen spots	48
		Throw away meat	25
		Overcook meat	13
		Normal use of meat	11
	What is your source of water?	Boiling	3
		Own well	27
		Borehole	59
		Communal	14

**Table 4:** Univariate analysis of bTB transmission to people in cattle owning households.

Factor	*p* Value	Odds Ratio (95% CI)
Weekly consumption of milk	0.31	0.24 (0.04–3.22)
Consumption of milk when needed	0.20	2.14 (0.70–6.74)
Purchase of meat from other farmers	0.21	0.37 (0.06–1.70)
bTB positive herd	<0.01	10.8 (2.97–51.08)

**Table 5:** Significant risk factors for bTB transmission to people that live in the households that own cattle from logistic regression.

Factor	*p* Value	Adjusted OR	CI_95%_
Bovine TB positive cattle	0.00009 ***	29.308	6.495–208.361
Consumption of milk when needed	0.0393 *	4.937	1.197–27.130
Consumption of meat from own cattle	0.0493 *	0.053	0.001–0.755
Consumption of beef bought from other farmers	0.0674	0.105	0.005–0.866
Awareness of bTB transmission	0.0471 *	4.419	1.101–22.067

Notes: * *p* < 0.05 *** *p* < 0.001.
